# Supplementary material for: Exciton dissociation in 2D layered metal-halide perovskites
Source: Nat Commun. 2023 Jul 11;14:4125. doi: 10.1038/s41467-023-39831-5 (PMC10336065; doi:10.1038/s41467-023-39831-5)
Supplement: Supplementary file 1 — Supplementary Information [file 41467_2023_39831_MOESM1_ESM.pdf]

Exciton dissociation in 2D layered metal-halide perovskites

SUPPLEMENTARY INFORMATION

Angelica Simbula[1,*], Luyan Wu[1], Federico Pitzalis[1], Riccardo Pau[1,2], Stefano Lai[1], Fang Liu[3], Selene Matta[1], Daniela Marongiu[1], Francesco Quochi[1], Michele Saba[1,*], Andrea Mura[1], Giovanni Bongiovanni[1]

[1]Dipartimento di Fisica, Università degli Studi di Cagliari, Monserrato (CA), I-09042, Italy
[2] Zernike Institute for Advanced Materials, University of Groningen, Nijenborgh 4,09747 AG Groningen, The Netherlands
[3] School of Environmental Science and Engineering, Frontiers Science Center for Transformative Molecules, Shanghai Jiao Tong University, Shanghai 200240, China
*Corresponding authors. e-mail angelica.simbula@unica.it, saba@unica.it

Supplementary Table 1 | Refined unit cell parameters of PEA_2PbI_4 single crystal. Crystal data and structure refinement for PEA_2PbI_4 recorded at 293 K on a Bruker D8 Venture diffractometer equipped with an Incoated microsource (Mo *K*α, λ = 0.71073 Å) and a PHOTON II detector. Layered structure is in agreement with the crystal structure deposited on the CCDC with refcode: BARHOU (ref. DOI: 10.1007/s12274-016-1401-6).

**Compound**<br>**Empirical formula**	**PEA_2PbI_4**<br>**$C_{16}H_{24}I_4N_2Pb$**
**Crystal system**<br>**Space group**	Triclinic<br>$P\bar{1}$
**a (Å)**<br>**b (Å)**<br>**c (Å)**	8.680(3)<br>8.682(3)<br>16.418(6)
**α (°)**<br>**β (°)**<br>**γ (°)**	94.512(13)<br>100.618(19)<br>90.559(12)
**Volume (Å³)**	1212.0(11)

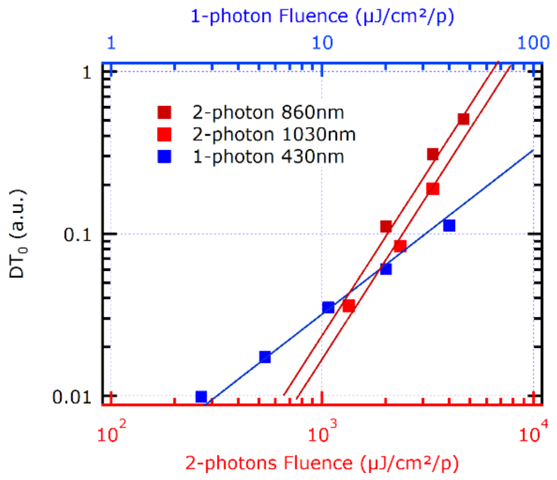

Supplementary Fig. 1 | DT intensity of thin films at time delay t=0 versus excitation fluence. Differential transmission bleaching intensity at time delay zero versus excitation fluence, as measured from a thin crystal. One-photon non-resonant is shown in blue, two-photon non-resonant in red and two-photon resonant in amaranth red. The Blue and red lines are a guide to the eye for linear and quadratic trend, respectively.

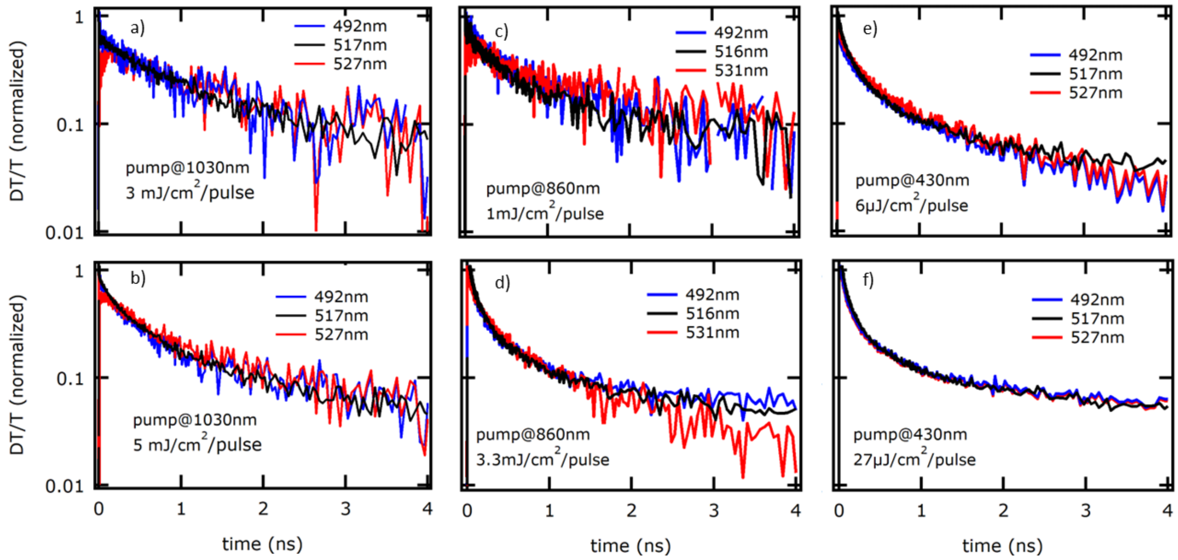

Supplementary Fig. 2 | DT/T spectral features decay profiles of thin film with different excitation condition. DT/T decay profiles (in absolute value) obtained from thin film with two-photon resonant (a-b), two-photon non-resonant (c-d) and one-photon non-resonant (e-f) excitation, reported for two different excitation fluences. The traces are obtained integrating the positive peak at central wavelength (black lines), negative lobe at longer wavelength (red lines) and at shorter wavelength (blue lines).

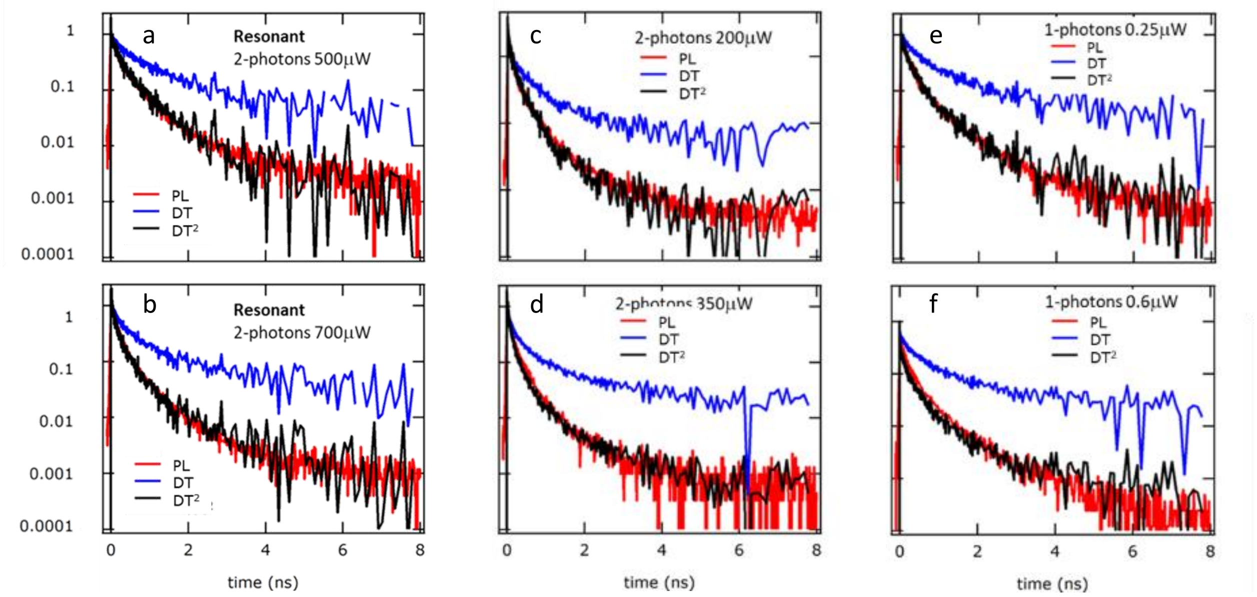

Supplementary Fig. 3 | DT/T, DT/T^2 and PL decay profiles for thin film at different power and excitation condition. Comparative plot with DT/T (blue lines), DT/T^2 (black lines) and PL (red lines) from thin films showing that in different excitation conditions the PL decay overlaps with that of DT/T^2 in the case of two-photon resonant (a-b), two-photon non-resonant (c,d) and one-photon non-resonant (e,f) excitation.

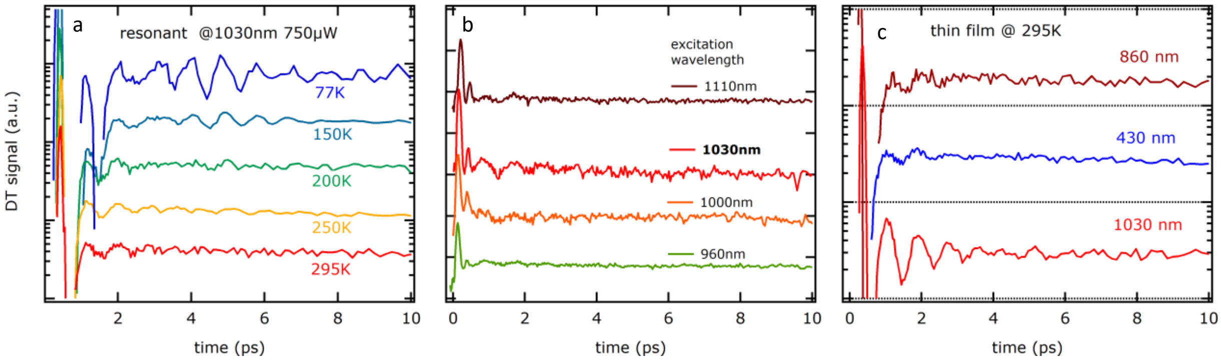

Supplementary Fig. 4 | Initial time oscillations of DT/T at different temperature and excitation wavelength. Initial time decay of DT signal measured on crystals with **a** 2-photons resonant excitation at fixed power, as a function of temperature from 77 K to 295K, and **b** at 295 K at different excitation wavelengths around the 2-photons resonant excitation energy. **c:** DT signal measured in thin films at room temperature, with the three explored excitation regimes. Oscillations are characterized by a constant period of about one picosecond, compatible with the Raman mode of PEA_2PbI_4 (30 cm^{-1}), not depending on the excitation wavelength, an amplitude that is maximized in the case of resonant excitation. In principle, oscillation may occur also in the excitonic/polaronic excited state, but a dependance on the excess energy would be expected. The reported phenomenology allows us to exclude that oscillation of the excited state are happening, while it is well compatible with coherent phonon oscillations in the fundamental state, triggered by a Raman process occurring during the pump pulse.

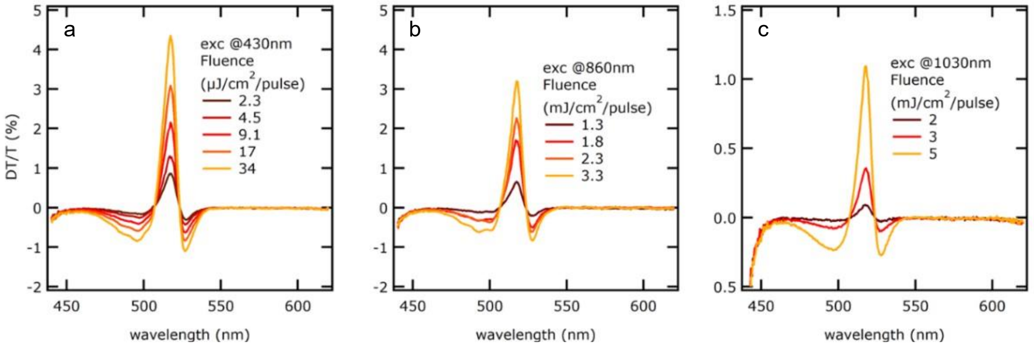

Supplementary Fig. 5 DT/T spectra of thin films after initial transient - DT spectra integrated after 40 ps at different laser fluences for three different excitation configurations: a) one-photon non-resonant, b) two-photon non-resonant and c) two-photon resonant. The spectra are confirming that after the initial ultrafast transient the behavior is always compatible with broadening feature, as sketched in Fig. 2c in main text.

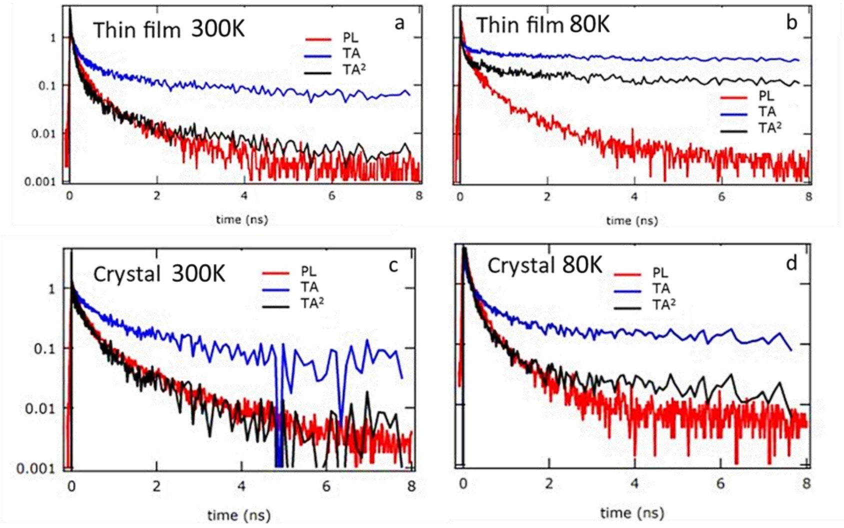

Supplementary Fig. 6 | DT/T, DT/T^2 and PL decay profiles for thin film and thin crystals at different temperature. Comparative plot with DT/T (blue lines), DT/T^2 (black lines) and PL (red lines) on thin film (a, b) and crystal (c, d) at different temperatures. In the case of thin film, deviation from the correspondence between PL and DT/T^2 appear at low temperature, which can be attributed traps, likely due to grain boundaries and surface defects. In the case of low temperature measurements in the crystal, the correspondence between PL and DT/T^2 is confirmed even at 80 K, as a sign of the lower defect amount in the crystal.

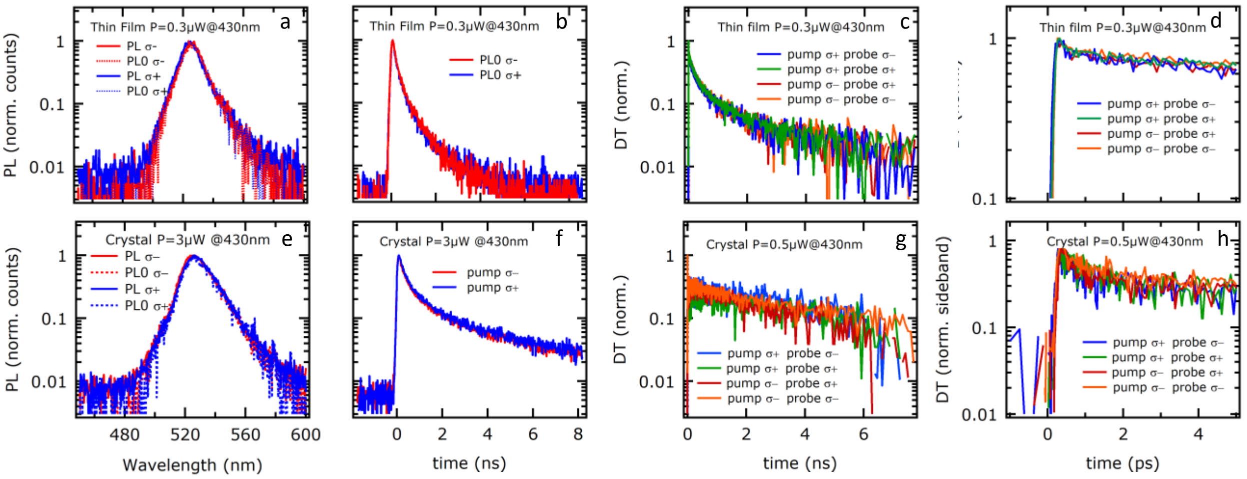

Supplementary Fig. 7 | Polarization dependent DT/T and PL measurements. Results of measurements of TRPL and DT measured on thin films (**a-d**) and on crystals (**e-h**) with circularly polarized pulsed laser with one-photon non-resonant excitation. Both PL spectra at time 0 and spectra integrated on a long delay are not depending on the sign of circular polarization (**a** and **e** thin film and crystal respectively), as well as the PL decay time (**b** and **f** thin film and crystal respectively). Time profile of DT bleaching signal in the thin film (**c** and **d** ns and ps timescale respectively) and of DT negative sideband in the crystal (**g** and **h** ns and ps timescale respectively) are also not visibly altered by circular polarization being positive or negative. Differences between crystal and film are mainly due to reabsorption, affecting the higher energy part of PL spectra, and to a higher quality of the crystal with respect to thin film, which reflects in a longer lifetime of both PL and DT.

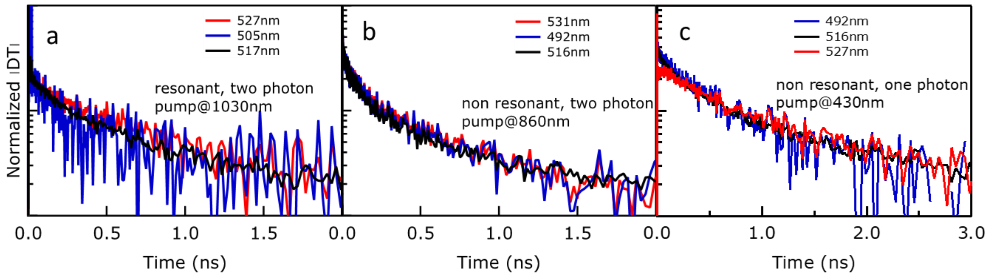

Supplementary Fig. 8 | DT/T spectral features decay profiles of crystal on nanosecond timescale. DT/T decay profiles (in absolute value) obtained from thin crystal with two-photon resonant (a), two-photon non-resonant (b) and one-photon non-resonant (c) excitation, on a nanosecond timescale, from the same set of data as what reported in Fig. 3 in the main text. The traces are obtained integrating the positive peak at central wavelength (black lines), negative lobe at longer wavelength (red lines) and at shorter wavelength (blue lines).

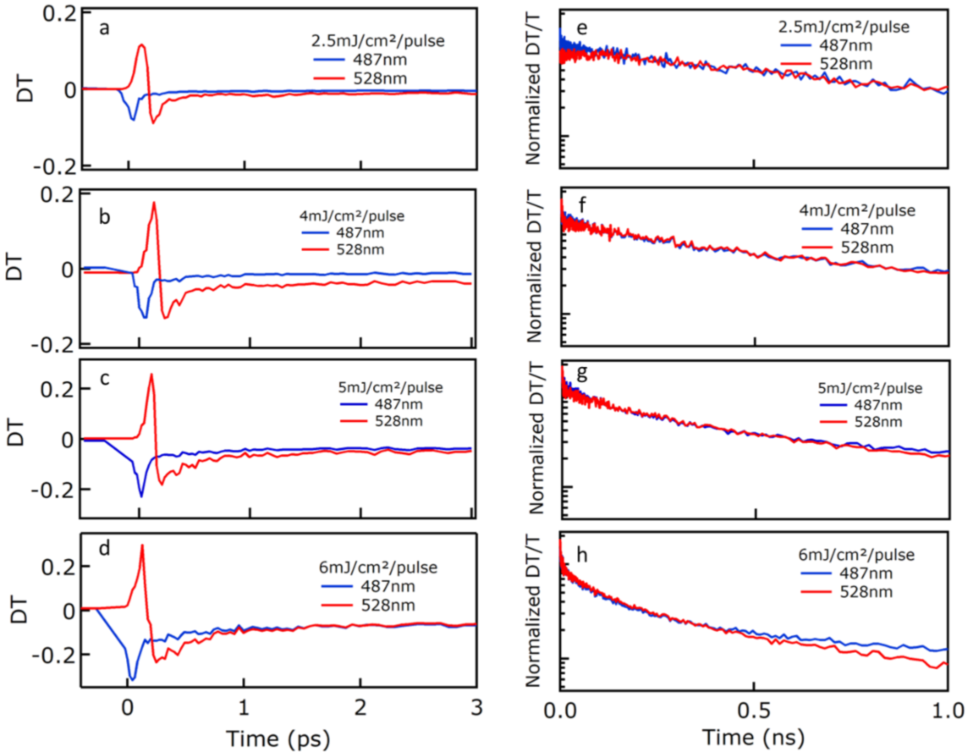

Supplementary Fig. 9 | DT/T decay profiles from thin crystal with two-photon resonant excitation. DT/T decay profiles obtained from thin crystal with two-photon resonant excitation (1030 nm) at the high energy lobe (487 nm) and low energy lobe (528 nm), reported with blue and red line respectively. Left side plots (a-d) report the picosecond scale initial transient, while right side plots (e-h) report the normalized absolute values of decay profiles on a log scale, on a nanosecond timescale. Profiles are extracted at different excitation fluences.

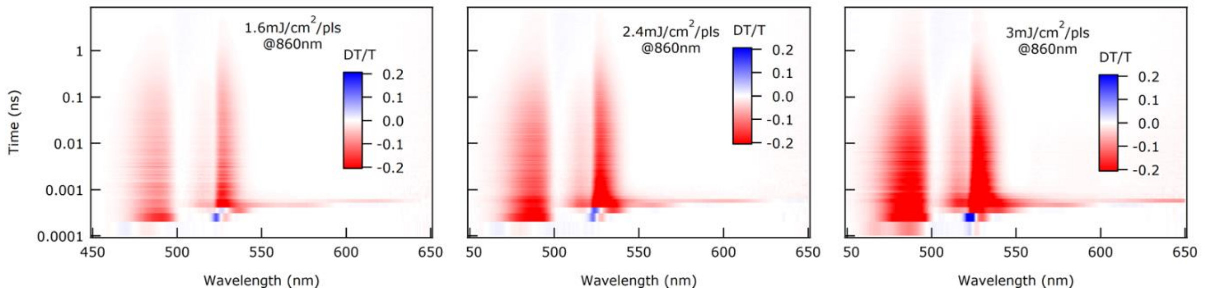

Supplementary Fig. 10 | DT/T spectrograms of thin crystals under two-photon non-resonant excitation. DT/T spectrograms of PEA_2PbI_4 thin crystals under two-photon non-resonant (860 nm) excitation at 300 K at different excitation fluences.

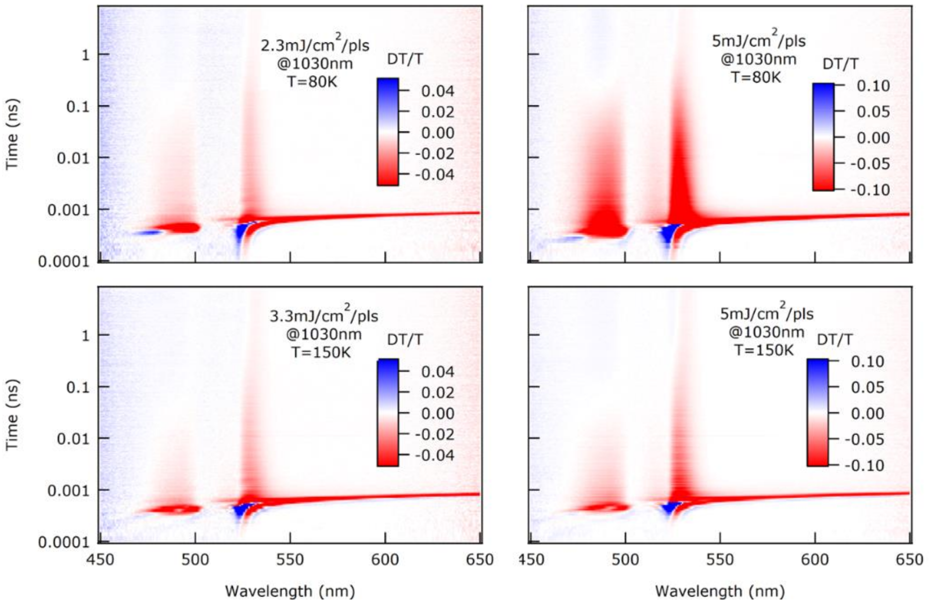

Supplementary Fig. 11 | DT/T spectrograms under two-photon resonant excitation at low T. DT/T spectrograms of PEA_2PbI_4 thin crystals under two-photon resonant excitation at low temperatures (upper plots at 80 K, lower plots at 150 K) at two different fluences, showing a trend similar to what reported at 300 K.

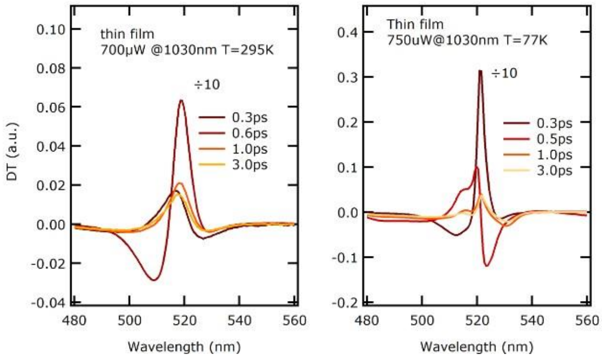

Supplementary Fig. 12 | DT/T initial transient at room and at cryogenic temperature. Comparison between the initial ultrafast dynamics of DT signal measured on thin film with 2-photon resonant excitation at different temperatures. The dynamics at cryogenic temperature is comparable to what measured at room temperature, suggesting that spontaneous polaronic deformation is not noticeably affected by temperature. To be noticed that in both cases the signal at 0.3 ps, where the intense negative band on the high-energy side is well visible, was reduced by a factor 10 to make it comparable with the signal at subsequent time delay.

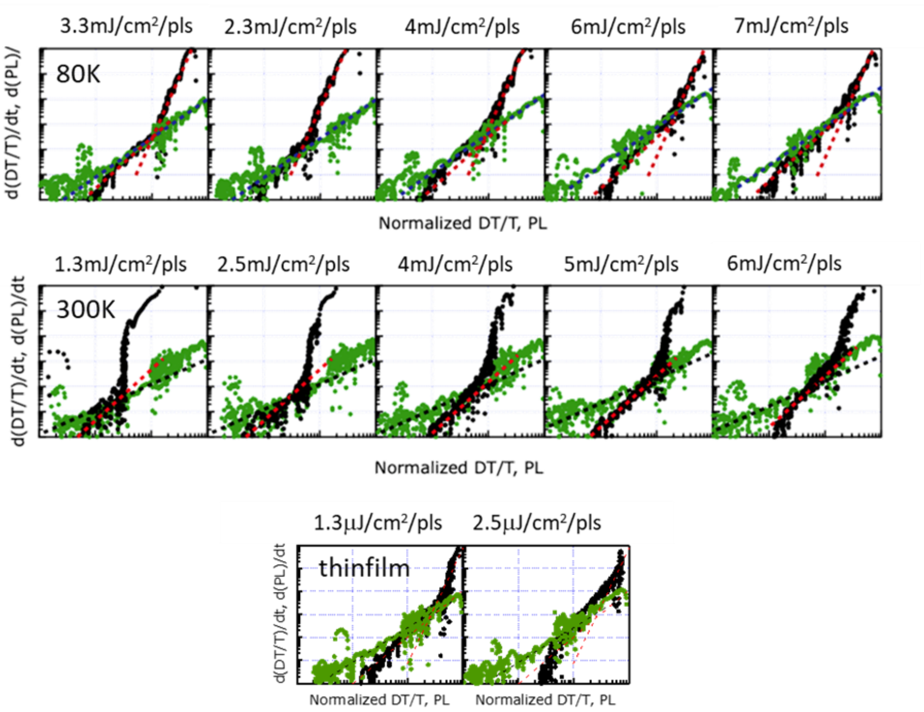

Supplementary Fig. 13 | DT/T and PL power laws at cryogenic and room temperature. Time derivative of DT/T versus DT/T (black dots) and time derivative of PL versus PL (green dots). Upper row is for thin crystal at 80 K, mid row for thin crystal at 300 K, lower row for thin film at 300 K, with two-photon resonant excitation, at difference fluences.

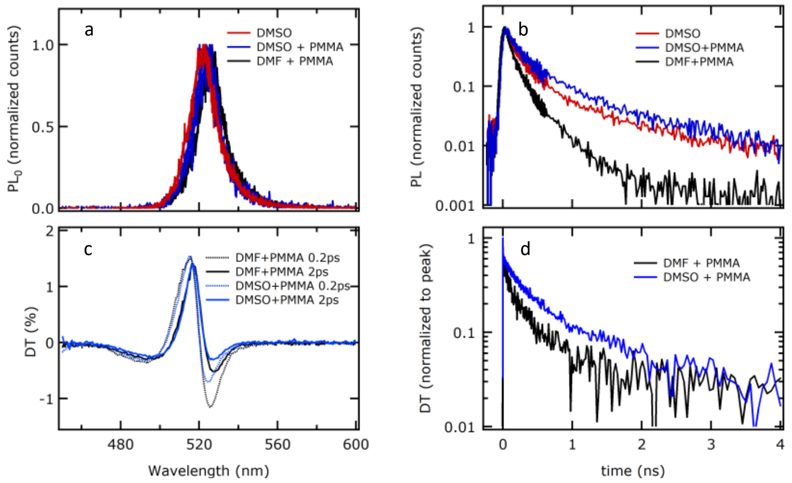

Supplementary Fig. 14 | DT/T and PL spectra and decays on thin films from different solvent and effect of PMMA. Plots of PL spectra **a**, PL time decays **b,** DT spectra at different time in the initial transient **c** and DT time decays **d,** obtained at the same excitation power (about 1 μJ/cm^2/p, at 430 nm) for different samples: PEA_2PbI_4 thin film from DMSO-based solution without (red) and with (blue) PMMA coating, compared to that of DMF-based solution (black), with PMMA coating. DT data from samples without PMMA is not reported due to degradation of the sample on a timescale comparable with the duration of a DT measurement (typically 15-30 minutes). Thin films covered with PMMA show better stability, in the considered excitation range (below 10 μJ/cm^2/pulse at 430 nm excitation wavelength) allowing to perform multiple measurements without evident signs of alteration. Decay time of DMSO-based sample is on the order of a fraction of ns (around 0.2 ns), compatibly with what typically found in literature, and in our case with a better performance with respect to DMF-based sample. DT and PL spectra are not noticeably affected by the chosen solvent, while the difference in both PL and DT lifetimes shows that the DMSO-based thin film has longer lifetimes, suggesting a higher quality.
